# Supplementary material for: Causal Relationship between Aging and Anorexia Nervosa: A White-Matter-Microstructure-Mediated Mendelian Randomization Analysis
Source: Biomedicines. 2024 Aug 16;12(8):1874. doi: 10.3390/biomedicines12081874 (PMC11351342; doi:10.3390/biomedicines12081874)
Supplement: Supplementary file 1 [file biomedicines-12-01874-s001.zip › Supplementary Figure/Figure S4/Singe SNP plot of MR analysis for TL to ubm-b-1815.pdf]

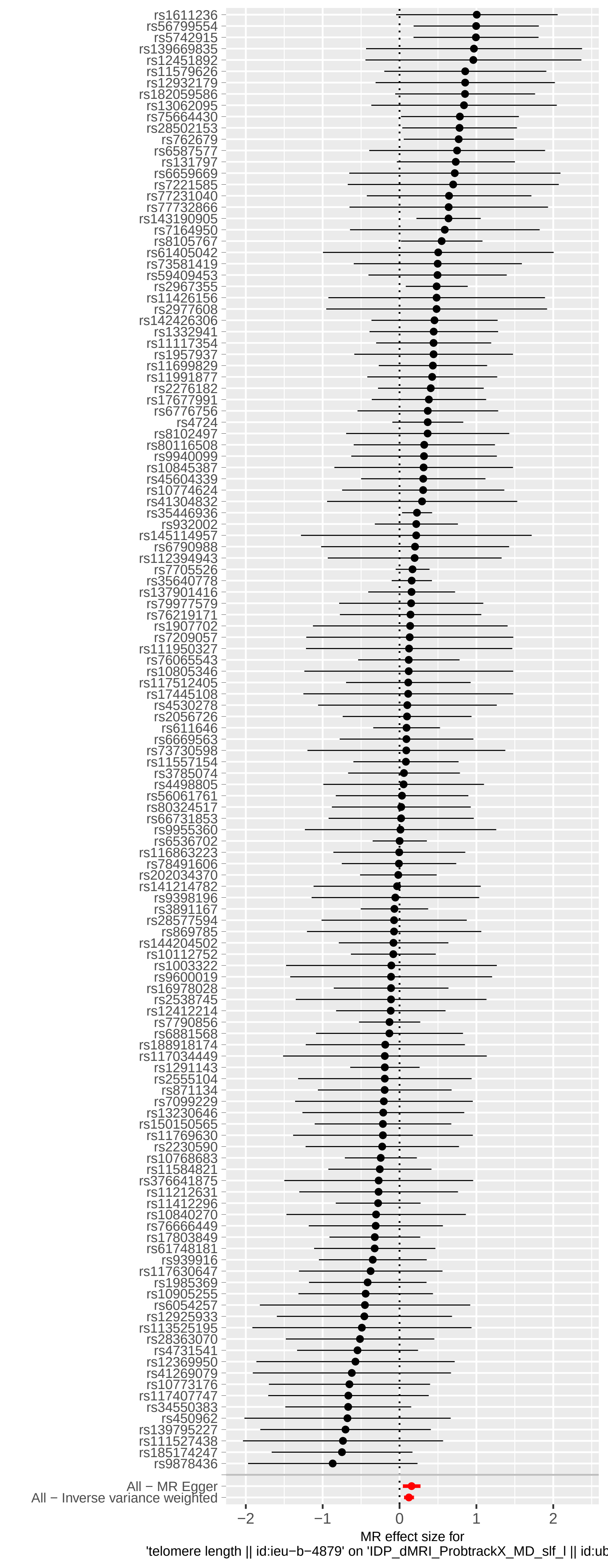
rs1611236
rs56799554
rs5742915
rs139669835
rs12451892
rs11579626
rs12932179
rs182059586
rs13062095
rs75664430
rs28502153
rs762679
rs6587577
rs131797
rs6659669
rs7221585
rs77231040
rs77732866
rs143190905
rs7164950
rs8105767
rs61405042
rs73581419
rs59409453
rs2967355
rs11426156
rs2977608
rs142426306
rs1332941
rs11117354
rs1957937
rs11699829
rs11991877
rs2276182
rs17677991
rs6776756
rs4724
rs8102497
rs80116508
rs9940099
rs10845387
rs45604339
rs10774624
rs41304832
rs35446936
rs932002
rs145114957
rs6790988
rs112394943
rs7705526
rs35640778
rs137901416
rs79977579
rs76219171
rs1907702
rs7209057
rs111950327
rs76065543
rs10805346
rs117512405
rs17445108
rs4530278
rs2056726
rs611646
rs6669563
rs73730598
rs11557154
rs3785074
rs4498805
rs56061761
rs80324517
rs66731853
rs9955360
rs6536702
rs116863223
rs78491606
rs202034370
rs141214782
rs9398196
rs3891167
rs28577594
rs869785
rs144204502
rs10112752
rs1003322
rs9600019
rs16978028
rs2538745
rs12412214
rs7790856
rs6881568
rs188918174
rs117034449
rs1291143
rs2555104
rs871134
rs7099229
rs13230646
rs150150565
rs11769630
rs2230590
rs10768683
rs11584821
rs376641875
rs11212631
rs11412296
rs10840270
rs76666449
rs17803849
rs61748181
rs939916
rs117630647
rs1985369
rs10905255
rs6054257
rs12925933
rs113525195
rs28363070
rs4731541
rs12369950
rs41269079
rs10773176
rs117407747
rs34550383
rs450962
rs139795227
rs111527438
rs185174247
rs9878436
All − MR Egger
All − Inverse variance weighted
−2
−1
0
1
2
MR effect size for
'telomere length || id:ieu−b−4879' on 'IDP_dMRI_ProbtrackX_MD_slf_l || id:ub [illegible]
